# Meisoindigo Protects Against Focal Cerebral Ischemia-Reperfusion Injury by Inhibiting NLRP3 Inflammasome Activation and Regulating Microglia/Macrophage Polarization via TLR4/NF-κB Signaling Pathway

**DOI:** 10.3389/fncel.2019.00553

**Published:** 2019-12-16

**Authors:** Yingze Ye, Tong Jin, Xu Zhang, Zhi Zeng, Baixin Ye, Jinchen Wang, Yi Zhong, Xiaoxing Xiong, Lijuan Gu

**Affiliations:** ^1^Central Laboratory, Renmin Hospital of Wuhan University, Wuhan, China; ^2^Department of Anesthesiology, Renmin Hospital of Wuhan University, Wuhan, China; ^3^Department of Neurosurgery, Renmin Hospital of Wuhan University, Wuhan, China; ^4^Department of Pathology, Renmin Hospital of Wuhan University, Wuhan, China; ^5^Department of Hematopathology, Renmin Hospital of Wuhan University, Wuhan, China

**Keywords:** ischemic stroke, NLRP3 inflammasome, microglia/macrophage polarization, toll-like receptor 4, new therapies

## Abstract

Ischemic stroke is a devastating disease with long-term disability. However, the pathogenesis is unclear and treatments are limited. Meisoindigo, a second-generation derivative of indirubin, has general water solubility and is well-tolerated. Previous studies have shown that meisoindigo reduces inflammation by inhibiting leukocyte chemotaxis and migration. In the present study, we investigated the hypothesis that meisoindigo was also protective against ischemic stroke, then evaluated its underlying mechanisms. *In vivo*, adult male C57BL/6J wild-type mice were used to produce a middle cerebral artery occlusion (MCAO) stroke model. On day three after reperfusion, obvious improvement in neurological scores, infarct volume reduction and cerebral edema amelioration were observed in meisoindigo treatment. Moreover, immunofluorescence staining and western-blot showed that the expression of NLRP3 inflammasome and its associated proteins in neurons and microglia was inhibited by meisoindigo. The effects of Meisoindigo on NLRP3 inflammasome inactivation and increased the M2 phenotype of microglia/macrophage through shifting from a M1 phenotype, which was possibly mediated by inhibition of TLR4/NF-κB. Furthermore, we verified the inhibitory effect of meisoindigo on TLR4/NF-κB signaling pathway, and found that meisoindigo treatment could significantly suppressed the expression of TLR4/NF-κB pathway-associated proteins in a dose-dependent manner, meanwhile, which resulted in downregulation of HMGB1 and IL-1β. Next, we established an *in vitro* oxygen glucose deprivation/Reperfusion (OGD/R) model in HT-22 and BV2 cells to simulate ischemic conditions. Cytotoxicity assay showed that meisoindigo substantially improved relative cell vitality and in HT-22 and BV2 cells following OGD/R *in vitro*. After suffering OGD/R, the TLR4/NF-κB pathway was activated, the expression of NLRP3 inflammasome-associated proteins and M1 microglia/macrophage were increased, but meisoindigo could inhibit above changes in both HT-22 and BV2 cells. Additionally, though lipopolysaccharide stimulated the activation of TLR4 signaling in OGD/R models, meisoindigo co-treatment markedly reversed the upregulation of TLR4 and following activation of NLRP3 inflammasome and polarization of M1 microglia/macrophages mediated by TLR4. Overall, we demonstrate for the first time that meisoindigo post-treatment alleviates brain damage induced by ischemic stroke *in vivo* and *in vitro* experiments through blocking activation of the NLRP3 inflammasome and regulating the polarization of microglia/macrophages via inhibition of the TLR4/NF-κB signaling pathway.

## Introduction

Ischemic stroke is a chief cause of disability and death worldwide (Donnan et al., 2008), but present therapies are limited. There is mounting evidence that the inflammatory response plays complex and multiphasic roles in the pathogenesis and progression of ischemic stroke. Clinically, the susceptibility and prognosis of stroke is also related to systemic inflammatory processes (Dirnagl et al., 1999). Although anti-inflammatory agents have garnered considerable interest from researchers for treating ischemic damage, and have exhibited promising effects in ischemic stroke animal models, most have failed in subsequent clinical trials (Guekht et al., 2017). Thus, new therapies that aim to re-establish brain homeostasis and restore the balance between pro-inflammatory and anti-inflammatory signals are urgently needed to improve outcome.

Indirubin (3,2′-bisindole), an active ingredient of the traditional Chinese herbal medicine *Dangui Luhui Wan*, is currently applied to treat chronic myeloid leukemia (CML) by inducing apoptosis, cell differentiation, and cell cycle arrest (Zhao et al., 2019). Indirubin also undertakes functions of anti-bacteria, anti-virus and anti-inflammation (Gaboriaud-Kolar et al., 2015; Memari et al., 2015; Gao et al., 2016). However, indirubin has poor water solubility and severe gastrointestinal side effects in some patients (Zheng et al., 1979). Meisoindigo, a 3,3′-linked bisindole, is a second-generation derivative of indirubin that is similar in structure to the parent. Meisoindigo is significantly more resistant to protein kinases and exhibits higher antitumor activity than indirubin. Meisoindigo has numerous biological effects, including anti-neoplastic (Cheng et al., 2016), anti-inflammatory (Zhang et al., 2013) and anti-leukocyte chemotactic effects (Ye et al., 2017). However, whether meisoindigo exerts a neuroprotective action in ischemic stroke remains unknown.

Microglia, as the brain resident macrophages, are the first line of defense against ischemic stroke (Hanisch and Kettenmann, 2007). After ischemic stroke, the resident microglia and circulating macrophages are activated and recruited to the damaged area to clear debris and initiate reparative processes (Zhao et al., 2015; Cai et al., 2018). Interestingly, the activated microglia/macrophages exert dual contrasting effects on CNS injury and recovery, which are related to their two distinct phenotypes (Hu et al., 2012; Liu et al., 2013; Hamzei Taj et al., 2016). Numerous studies show that microglia/macrophages are highly plastic cells (Kigerl et al., 2009; Cai et al., 2018) that can acquire diverse phenotypes and functions in response to specific cues. Classically-activated macrophages (M1) produce pro-inflammatory cytokines, such as CD16, CD32, CD86 and inducible nitric oxide synthase (iNOS), which are considered detrimental to recovery (Patel et al., 2013; Wang et al., 2018). In contrast, anti-inflammatory mediators, including interleukin (IL)-4, IL-10, IL-13 and transforming growth factor-β (TGF-β), which are believed to be beneficial, are synthesized by alternatively activated macrophages (M2) (Wang et al., 2018). Accumulating evidence indicates that microglia/macrophages phenotypically respond in a dynamic manner during the progression of ischemic stroke (Hu et al., 2012). They display a “sick” M1 phenotype after a transient “healthy” M2 phenotype (Hu et al., 2012). However, the effect of the polarization status of microglia/macrophages in stroke and the associated inflammatory signaling are still unclear.

Recently, the inflammasome protein complex has been recognized as a crucial player in the innate immune response during ischemic stroke (Fann et al., 2013). There are four types of inflammasomes in the CNS, including NOD-like receptor pyrin domain-containing protein 1 (NLRP1), NLRP3, NLR family CARD domain-containing protein 4 (NLRC4) and absent in melanoma-2 (AIM2). Among these, NLRP3 is the most extensively studied in CNS diseases (Walsh et al., 2014). There are two processions for NLRP3 inflammasome activation. Firstly, it needs to be primed by activating TLR4/NF-κB signaling pathway to promote the transcription of NLRP3 components (Kopitar-Jerala, 2015). Then, NLRP3 protein forms a complex with apoptosis-associated speck-like protein containing a CARD (ASC), and then binds to the cysteine protease caspase-1 to form the inflammasome. This leads to activation of caspase-1 that shears pro-IL-1β and pro-IL-18 to their mature (IL-1β and IL-18) forms, which mediate inflammatory responses or initiate the process of inflammatory cell death—pyroptosis (Schroder and Tschopp, 2010; Coll et al., 2015). The novel compound MCC950, an inhibitor of the NLRP3 inflammasome, has been shown to play a neuroprotective role via specific inhibition of NLRP3-induced ASC oligomerization in stroke (Ismael et al., 2018). Some studies have reported that the NLRP3 inflammasome is involved in the activation of M1 microglia after stroke (Ji et al., 2017), and the conversion processes of procaspase-1 to caspase-1 also contributes to the production and secretion of M1 typed mature pro-inflammatory cytokines (Slusarczyk et al., 2018). However, the function of NLRP3 in phenotypic polarization of microglia/macrophages is unclear. The links between NLRP3 inflammasome activation, microglia/macrophage polarization and stroke also need to be elucidated. In addition, the impact of meisoindigo on NLRP3 inflammasome activation and microglia/marophages polarization is also unknown.

The Toll-like receptor (TLR) is a pattern-recognition receptor that detects microbial components, and plays an important role in the initiation of the immune response (Liu et al., 2019). TLR4, which is expressed in neuron, microglia and astrocytes in the brain, recognizes lipopolysaccharide (LPS) and is involved in the release of inflammatory mediators by activating the NF-κB signaling pathway. Studies have demonstrated that TLR4-deficient/knockout mice have minor infractions and less inflammatory response after an ischemic insult (Caso et al., 2007). A recent study indicates that meisoindigo was also found to downregulate the TLR4–TAK–NF-κB pathway, which played critical roles in inflammatory cytokines release (Zhang et al., 2013). In addition, we previously showed that meisoindigo inhibits leukocyte chemotactic migration, which plays a critical role in the inflammatory response (Ye et al., 2017). These observations led us to conjecture that meisoindigo might regulate the immune response via TLR4/NF-κB mediated inflammatory signaling following stroke.

In this study, we first examined whether meisoindigo exerted a neuro-protective effect in a mouse MCAO model and *in vitro* OGD/R models. We then examined whether meisoindigo impacted NLRP3 inflammasome activation and M1–M2 shift after stroke, and whether TLR/NF-κB signaling pathway participated in the anti-inflammation and neuro-protective effect of meisoindigo. Next, we used *in vitro* oxygen glucose deprivation (OGD) models in HT-22 cells and BV2 cells to confirm those above effects of meisoindigo and the underlying TLR/NF-κB signaling pathway against cerebral ischemia reperfusion injury (CIRI) by co-treatment with a combination of meisoindigo and LPS Our results showed that meisoindigo may protect against cerebral ischemic injury in the brain by suppressing NLRP3 inflammasome activation and M1 polarization via inhibiting TLR/NF-κB signaling pathway, which is expected to be a promising new drug candidate for the treatment of ischemic stroke.

## Materials and Methods

### Animals

Wild-type C57BL/6J mice (*n* = 130, by excluded the death animals and unsuccessful models including without infarction or infarction with hemorrhage, 25–30 g) were purchased from Hunan Silaikejingda (SJA) Laboratory Animal, Co. (Changsha, China; Nos. 43004700018817, 43004700020932). All animal experimental protocols were approved by the Animal Experimentation Ethics Committee of Wuhan University (No. WDRM-20170504) and were conducted according to the Animal Care and Use Committe guidelines of Renmin Hospital of Wuhan University. Animals were housed in a room with controlled humidity (65 ± 5%) and temperature (25 ± 1°C), under a 12/12-h light/dark cycle with free access to food and water for at least 1 week before the experiments.

### Drug Administration and Experimental Groups

Meisoindigo (100 mg; #97207-47-1, National Institutes for Food and Drug Control, Beijing, China) was dissolved in dimethyl sulfoxide, and then diluted with sterile saline to the desired concentrations. Before MCAO and 2 h after reperfusion, different concentrations of meisoindigo were intraperitoneally (i.p.) administered to the animals. MCC950 (PZ0280, Sigma-Aldrich, St. Louis, MO, United States) was dissolved with physiological saline solution, and administered (50 mg/kg, i.p.) 1 and 3 h after occlusion (Coll et al., 2015; van Hout et al., 2017; Ismael et al., 2018). TAK-242 (HY-11109, MedChemExpress, Monmouth Junction, NJ, United States) was dissolved in dimethyl sulfoxide and then diluted in sterile saline. After 1 h occlusion, TAK-242 was injected (3 mg/kg, i.p.) and optimal dose was selected based on previous studies (Rice et al., 2010; Hua et al., 2015). The 110 mice were randomly allocated to the following eight groups (*n* = 15): sham operation, MCAO + vehicle, MCAO + meisoindigo (2 mg/kg), MCAO + meisoindigo (4 mg/kg), MCAO + meisoindigo (8 mg/kg), MCAO + meisoindigo (12 mg/kg), MCAO + MCC950 (50 mg/kg), and MCAO + TAK-242 (3 mg/kg). The vehicle solution containing no meisoindigo, MCC950 and TAK-242 was administered to the vehicle group.

### MCAO Model

The MCAO model was produced as previously described (Xiong et al., 2015, 2016). In brief, C57BL/6J wild-type mice were anesthetized with 5% isoflurane in O_2_ by facemask, followed by ligation of the left middle cerebral artery with 6-0 monofilament (Doccol, Corp., Redlands, CA, United States). After 1 h of occlusion, the monofilament was removed to initiate reperfusion. A homeothermic heating pad was employed to monitor and stabilize the mice body temperature at 37 ± 0.5°C. The same procedure, but without monofilament ligation, was performed on sham-operated mice.

### Infarct Volume Measurement

Mice were deeply anesthetized and euthanized with an overdose of isoflurane and decapitated 3 days after MCAO (i.e., after 3 days of reperfusion). The brains were collected after transcranial perfusion by saline followed with 4% paraformaldehyde. Brain tissues were cut into 1-mm coronal sections, and then dipped in 2% 2,3,5-triphenyltetrazolium chloride (TTC) (17779, Sigma-Aldrich, United States) for staining. The infarct volume was measured and analyzed by a blinded observer using ImageJ v1.37 (NIH, Bethesda, MA, United States), as described previously (Gu et al., 2012; Xiong et al., 2016; Stary et al., 2017), then was normalized and presented as a percentage of the non-ischemic hemisphere to correct for edema (Ouyang et al., 2012; Stary et al., 2017).

### Assessment of Neurological Deficit

Neurological deficit scores were evaluated 3 days after MCAO as described previously (Han et al., 2009; Gu et al., 2012). The score ranged from 0 (without observable neurological deficit) to 4 (no spontaneous motor activity and loss of consciousness).

### Brain Water Content

Cerebral edema was assessed 3 days after reperfusion by the wet/dry method, as previously described (Hatashita et al., 1988). In brief, the brains were removed without perfusion, and the wet weights were measured on an electronic balance. Dry weights were measured after heating the specimens at 105°C for 24 h. The brain water content was calculated by using the following formula: (wet weight-dry weight)/wet weight × 100%.

### Immunofluorescence Staining

Immunofluorescence was performed as previously described (Xiong et al., 2016). Ischemic and sham-operated mice were euthanized and perfused with cold PBS, followed by fixation with 4% paraformaldehyde for 2 days. The brains were then prepared into paraffin sections. Before staining, the sections were de-paraffinized, rehydrated and antigen-retrieved, followed by 0.3% hydrogen peroxide treatment to quench endogenous peroxidase activity. Afterward, the slices were blocked with 0.1M PBS containing 5% fetal bovine serum and 0.3% Triton X for 1 h at room temperature. After washing, the slices were incubated at 4°C overnight with the following primary antibodies: anti-CD68 (1:200; MCA1957,AbD Serotec, Oxford, United Kingdom), anti-NLRP3 (1:200; ab4207, Cell Signaling Technology, Boston, MA, United States), anti-NeuN (1:200; ab104224, Abcam, Cambridge, United Kingdom), anti-MPO (1:100, GB11224, Servicebio, Wuhan, China), anti-YM1/2 (1:200; 60130, STEMCELL Technologies, Vancouver, BC, Canada) and anti-iNOS (1:200; ab49999, Abcam, Cambridge, United Kingdom). The slices were then rinsed and incubated with an Alexa 594-conjugated antibody (1:200 for NeuN, CD68, MPO, and iNOS; ANT030, Millipore, Billerica, MA, United States) or an Alexa 488-conjugated antibody (1:200 for NLRP3 and YM1/2; ANT024, Millipore, Billerica, MA, United States for 2 h at room temperature. After thorough rinsing, the nuclei were stained with DAPI (94010, Vector Laboratories, Burlingame, CA, United States). All slices were photographed using an automatic fluorescence microscope (BX63, Olympus Optical, Ltd., Tokyo, Japan). The number of immunoreactive cells in predefined areas were quantified using ImageJ software (Media Cybernetics, Inc., Rockville, MD, United States). Six different fields for each mouse and six mice for each group were counted. All counts were conducted by blinded observers.

### Real-Time RT-PCR

On day 3 after MCAO, the penumbral region of the ischemic hemisphere was processed in RNAiso Plus (#9109, TaKaRa, Shiga, Japan) and chloroform to extract total RNA. After detecting the concentration and purity of the extracted total RNA, 1 μg of total RNA was reverse-transcribed with the PrimerScript RT reagent Kit containing gDNA Eraser (RR820a, TaKaRa, Shiga, Japan); to remove genomic DNA. The resulting cDNA was mixed with synthetic primers (Beijing Genomics Institute) and SYBR Premix Ex Taq2 (RR047a, TaKaRa, Shiga, Japan) for PCR. The primer sequences were gained through PrimerBank^1^ and are listed in Table 1. The thermocycling parameters were used as follows: 50°C for 2 min, 95°C for 10 min, followed by 45 cycles of 95°C for 10 s, 60°C for 10 s and 72°C for 15 s. The values were normalized to the internal reference GAPDH. The expression of the target gene was presented as fold change from the sham control.

**TABLE 1:** Primers for RT-PCR.

**Genes**		**Primers (5′–3′)**
iNOS	Forward	GTTCTCAGCCCAACAATACAAGA
	Reverse	GTGGACGGGTCGATGTCAC
CD16	Forward	CAGAATGCACACTCTGGAAGC
	Reverse	GGGTCCCTTCGCACATCAG
CD32	Forward	ATGGGAATCCTGCCGTTCCTA
	Reverse	CCGTGAGAACACATGGACAGT
Ym1	Forward	CAGGTCTGGCAATTCTTCTGAA
	Reverse	GTCTTGCTCATGTGTGTAAGTGA
Arg1	Forward	CTCCAAGCCAAAGTCCTTAGAG
	Reverse	AGGAGCTGTCATTAGGGACATC
CD206	Forward	CTCTGTTCAGCTATTGGACGC
	Reverse	CGGAATTTCTGGGATTCAGCTTC
TNF-α	Forward	GACGTGGAACTGGCAGAAGAG
	Reverse	TTGGTGGTTTGTGAGTGTGAG
IL-1β	Forward	GCAACTGTTCCTGAACTCAACT
	Reverse	ATCTTTTGGGGTCCGTCAACT
GAPDH	Forward	AGGTCGGTGTGAACGGATTTG
	Reverse	TGTAGACCATGTAGTTGAGGTCA

### *In vitro* Cell Culture, Oxygen-Glucose Deprivation/Reperfusion

Mouse hippocampal (HT-22) and murine microglia (BV2) cell lines were obtained from China Center for Type Culture Collection (Wuhan, China), and cultured in Dulbecco’s modified Eagle’s medium (DMEM) which was supplemented with 10% fetal bovine serum (FBS, #13011-8611, Zhejiang Tianhang Biotechnology, Co., Ltd., Zhejiang, China) and 1% antibiotic (GNM15140, Genome, China) in an incubator supplied with 5% CO_2_ at 37°C. Before inducing OGD/R injury, the cultured cells were in the logarithmic growth phase, and rinsed twice with PBS and maintained in glucose-free DMEM. Cells were then placed into a hypoxic incubator (Binder, CB-210 hypoxia workstation) with 1% O_2_, 5% CO_2_, and 94% N_2_ for suitable time at 37°C to mimic OGD injury. Cultures were then restored with glucose at DMEM and recovered at normoxic conditions (37°C, 5% CO_2_) for 12 h (OGD restoration), as described previously (Xiong et al., 2014). The control groups without OGD were washed twice with PBS, maintained in DMEM and no oxygen deprivation.

### Drug Treatment and Cell Viability Assay *in vitro*

Cell Counting Kit (CCK)-8 assay (Dojindo Laboratories, Kumamoto, Japan) was used to assess the cell viability. Briefly, neuronal cell line HT-22 and microglia cell line BV2 were seeded in 96-well plates with DMEM containment 10% FBS. Cells were treated with LPS (1 μg/mL) and different concentrations (10, 30, 50, 100, and 150 mM) of meisoindigo at the beginning of OGD. The optimal dose of LPS was selected based on previous studies (Chen et al., 2018). Then the medium was removed, 10 μl of CCK-8 solution was subsequently added to each well. After 2 h of incubation at 37°C, the absorbance at 450 nm was measured using an automatic microplate reader (PerkinElmer Victor 1420, Alburg, VT, United States).

### Western Blot Analysis

Western blotting was carried out as previously described (Xiong et al., 2015). Total protein was extracted from different conditions-treated HT-22 or BV2 microglia and ipsilateral brain tissue harvested 3 days after MCAO. Then, samples were homogenized in cold RIPA buffer (C1053, Applygen, Beijing, China) and added protease inhibitor cocktail (G2006, Servicebio, Wuhan, China). The homogenates were centrifuged at 4°C at 10,000 × *g* for 30 min, and then the supernatants were harvested. Protein content was determined with the BCA kit (G2026, Servicebio, Wuhan, China). Protein samples (20 μL/lane) were separated by electrophoresis on 4–15% sodium dodecyl sulfate-polyacrylamide gels and then transferred onto PVDF membranes (Millipore, Billerica, MA, United States). Membranes were then put into 5% non-fat milk with PBS/0.1% Tween and blocked for 1 h, followed by incubation overnight with mouse anti-NLPR3 (1:1,000; ab4207, Cell Signaling Technology, Boston, MA, United States), anti-ASC (1:1,000; 67824, Cell Signaling Technology, Boston, MA, United States), anti-CL-caspase-1 (1:500; 89332, Cell Signaling Technology, Boston, MA, United States), anti-IL-18 (1:500; ab207324, Abcam, Cambridge, United Kingdom), anti-TLR4 (1:1,000; ab8378, Abcam, Cambridge, United Kingdom), anti-NF-κB p65 (1:1,000; ab32536, Abcam, Cambridge, United Kingdom), anti-phosphorylated NF-κB p65 (1:1,000; 3033S, Cell Signaling Technology, Boston, MA, United States), anti-AQP4 (1:1,000; 2042744, Millipore, Billerica, MA, United States), anti-HMGB1 (1:1,000; ab18256, Abcam, Cambridge, United Kingdom) and anti-IL-1β (1:500; ab8320, Abcam, Cambridge, United Kingdom) at 4°C. After washing with PBS/0.1% Tween, the membrane was incubated with IRDye-labeled secondary antibody (1:10,000; c60405-05, Li-Cor Bioscience, United States) at room temperature for 1–2 h. Images were acquired with the Odyssey Western Blot Analysis system (LI-COR, Lincoln, NE, United States). The relative band intensity was calculated using Quantity One v4.6.2 software (Bio-Rad Laboratories, Hercules, CA, United States) and then normalized to the β-actin loading control. All above experiments were operated three times.

### Statistical Analysis

All data are presented as mean ± SD. One-way analysis of variance (ANOVA) followed by Tukey’s test for multiple comparisons was used to analyze statistical significance. *P* < 0.05 was regarded as statistically significant.

## Results

### Meisoindigo Reduces Infarct Volume and Ameliorates Neurological Deficits 3 Days After MCAO

**FIGURE 1:**
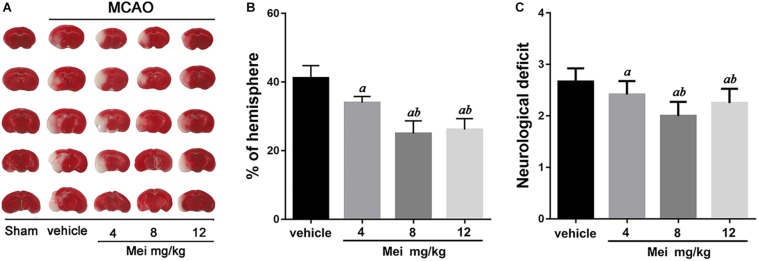
Meisoindigo injection reduces infarct size and ischemic stroke injury. **(A)** Infarct volume of the ipsilateral hemisphere was measured 3 days post-stroke onset by using TTC staining. **(B)** Ipsilateral Infarct size was normalized to the contralateral hemicerebrum and presented as a percentage. **(C)** Neurological scores. Mean ± SD. *n* = 5. *^a^P* < 0.05 vs. vehicle; *^b^P* < 0.05 vs. Mei 4 mg/kg.

To examine whether meisoindigo has a neuroprotective effect, we first constructed MCAO models, assessed infarct volume and neurobehavioral outcomes. Neurological scoring was performed 3 days after MCAO. Firstly, the result showed that there were some animals died in each group within 3 days post MCAO (the survival rate was shown in Supplementary Figure S1). Then, the survival mice were killed, and the brains were quickly isolated, sliced into 1 mm-thick coronal sections, and stained with TTC. Examination of all five brain slices showed that administration of meisoindigo (4, 8, and 12 mg/kg) significantly reduced infarct volume compared with the vehicle group (*P* < 0.05) (Figures 1A,B). Similarly, compared with the vehicle group, meisoindigo (4, 8 and 12 mg/kg) also alleviated the neurological deficits (*P* < 0.05) (Figure 1C). Additionally, the 8 mg/kg dose had the greatest protective effect, and was selected for subsequent experiments.

### Meisoindigo Reduces Edema and Lowers AQP4 Expression in the Brain 3 Days After MCAO

**FIGURE 2:**
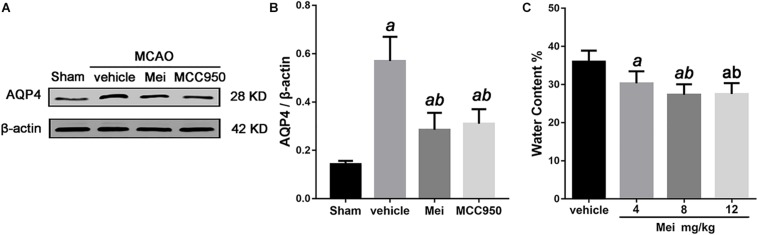
Meisoindigo injection reduces brain water content and the expression of AQP4 after ischemic stroke. **(A)** Western blot analysis showing that Mei treatment reduces the expression of AQP4. **(B)** Quantitative analysis of the protein levels of AQP4. **(C)** Quantification of water content 3 days after stroke. Mean ± SD. *n* = 5. *^a^P* < 0.05 vs. vehicle; *^b^P* < 0.05 vs. Mei 4 mg/kg; *^c^P* < 0.05 vs. Mei 8 mg/kg.

Cerebral ischemia causes brain edema, and the prognosis of ischemic stroke is related to the extent of the edema. AQP4, a water channel protein, plays a major role in the pathogenesis of cerebral edema. We therefore examined the effects of meisoindigo on brain edema and AQP4 expression after MCAO. Meisoindigo reduced brain water content and significantly downregulated the expression of AQP4 compared with the vehicle group (*P* < 0.05) (Figure 2). MCC950, a highly potent and selective inhibitor of the NLRP3 inflammasome, *in vitro* and *in vivo* (Ren et al., 2018; Stancu et al., 2019), had effects similar to those of meisoindigo.

### Meisoindigo Suppresses NLRP3 Inflammasome Activation After MCAO

**FIGURE 3:**
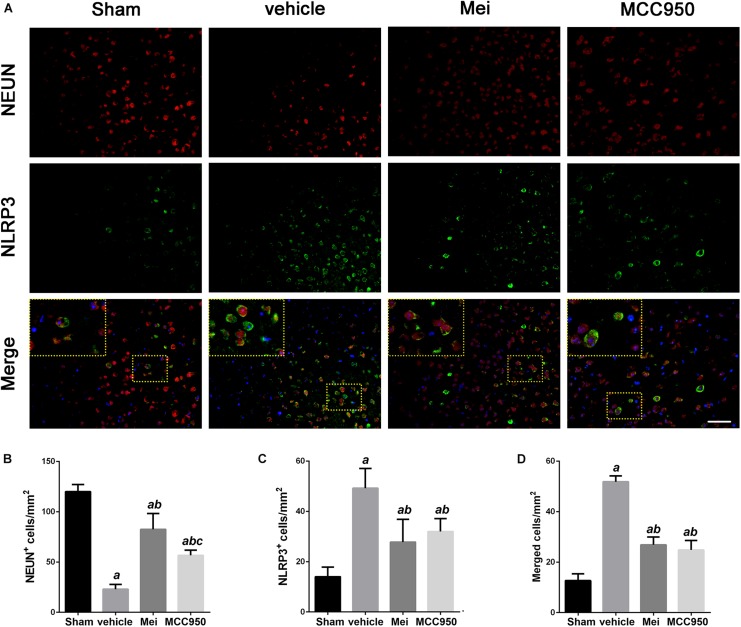
Meisoindigo injection reduces the number of NLRP3 and NeuN-positive cells in the penumbra region of the ischemic cortex. **(A)** Representative immunofluorescence images of NLRP3 and NeuN labeling, counterstained with DAPI, 3 days after ischemic stroke in the penumbra region. **(B,C)** Quantification of NeuN and NLRP3-positive cells in the ischemic penumbra. **(D)** Quantification of merged cells in the ischemic penumbra. Mean ± SD, *n* = 5. Scale bar = 50 μm. *^a^P* < 0.05 vs. sham; *^b^P* < 0.01 vs. vehicle; *^c^P* < 0.05 vs. Mei.

**FIGURE 4:**
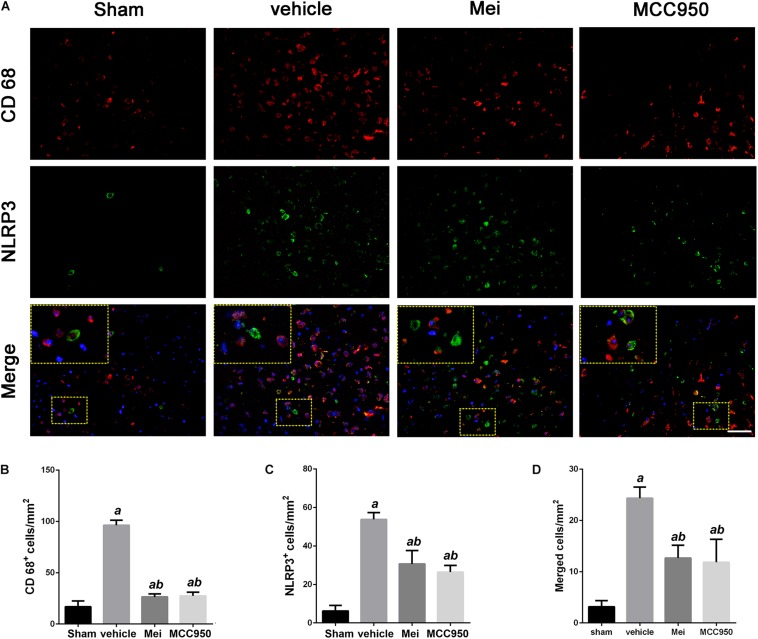
Meisoindigo injection reduces the number of NLRP3 and CD68-positive cells in the penumbra zone of the ischemic cortex. **(A)** Representative immunofluorescence images of NLRP3 and CD68 labeling, counterstained with DAPI, 3 days after ischemic stroke in the penumbra. **(B,C)** Quantification of CD68 and NLRP3-positive cells in the ischemic penumbra. **(D)** Quantification of merged cells in the ischemic penumbra. Mean ± SD, *n* = 5. Scale bar = 50 μm. *^a^P* < 0.05 vs. sham; *^b^P* < 0.05 vs. vehicle; *^c^P* < 0.05 vs. Mei.

**FIGURE 5:**
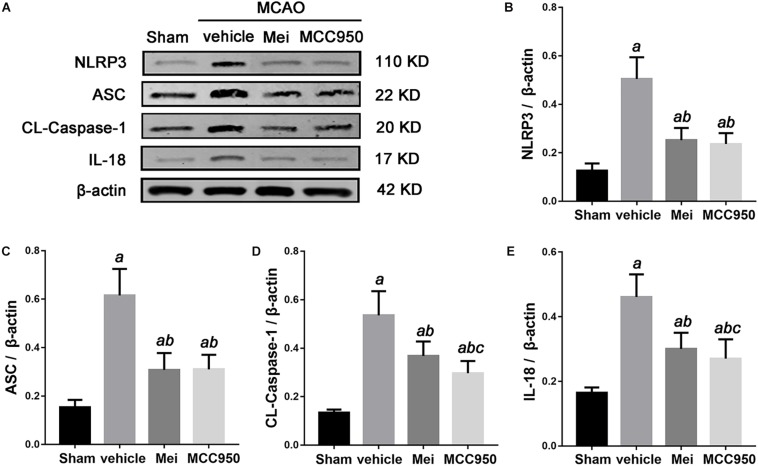
The effects of Meisoindigo on inflammasome proteins. **(A)** Western blot analysis showing that Mei treatment reduces the expression of NLRP3 inflammasome associated proteins, including NLRP3, ASC, CL-caspase-1 and IL-18, in the ischemic cortical tissue after 3 days of reperfusion. **(B–E)** Quantitative analysis of the protein levels of NLRP3, ASC, CL-caspase-1, and IL-18 in the ischemic cortical tissue. Mean ± SD, *n* = 5; *^a^P* < 0.05 vs. sham; *^b^P* < 0.05 vs. vehicle; *^c^P* < 0.05 vs. Mei.

To further investigate whether meisoindigo inhibits neuroinflammation after focal cerebral ischemia, we examined the expression of NLRP3 inflammasome-associated proteins, including NLRP3, ASC, CL-caspase-1 and IL-18, in the brain by immunofluorescence staining and western blotting. NLRP3^+^ cell counts showed that cerebral ischemia caused the activation of the NLRP3 inflammasome in the penumbra of the ischemic cortex (Figures 3A,C, 4A,C). Treatment with meisoindigo significantly resulted in a number reduction of NLRP3^+^ cells in the penumbra compared with the vehicle group (*P* < 0.05) (Figures 3A,C, 4A,C). Western blot analysis showed that meisoindigo reduced the levels of NLRP3, ASC, CL-caspase-1 and IL-18, and significant differences were found after 3 days of reperfusion compared with vehicle (*P* < 0.001) (Figure 5). To confirm the effect of meisoindigo on NLRP3 expression, we used the NLRP3 inflammasome inhibitor MCC950. The number of NLRP3^+^ cells was significantly reduced in the penumbra by MCC950, to an extent similar to that of meisoindigo (Figures 3A, 4C). Western blot analysis also confirmed that treatment with MCC950 markedly reduced the levels of NLRP3 inflammasome-associated protein, and have no statistical difference to compared with meisoindigo (*P* > 0.05) (Figure 5).

We also analyzed the number of neurons and microglia/macrophages in the ischemic penumbra, as neurons are important markers of neurological recovery and microglia/macrophages are key markers of neuroinflammation. Meisoindigo treatment significantly reduced neuronal loss (Figures 3A,B) and neuroinflammation (Figures 4A,B) in the ischemic penumbra 3 days after MCAO. Furthermore, NLRP3 immunoreactivity coincided more with NeuN^+^ neurons than with CD68^+^ microglia/macrophages (*P* < 0.05) (Figures 3D, 4D), and was negatively associated with NeuN staining and positively with CD68 staining, suggesting that NLRP3 may play a significant role in neuronal death (perhaps pyroptosis), and may relate to some inflammatory mediators released from not only infiltrated microglia/macrophages but also dying neurons in the penumbra.

### Meisoindigo Attenuates Neutrophil Infiltration and Modulates Microglia/Macrophage Polarization After MCAO

**FIGURE 6:**
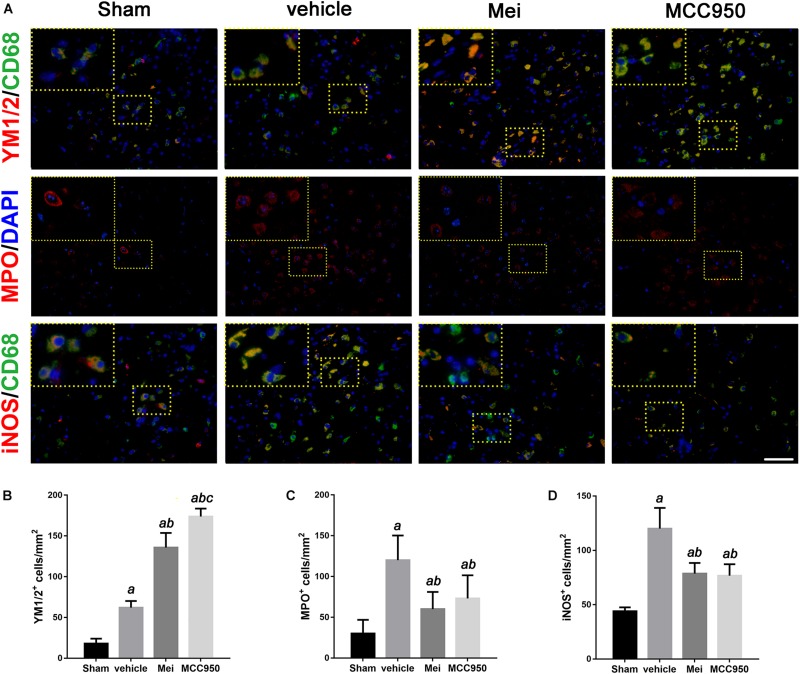
Meisoindigo injection reduces the number of YM 1/2, iNOS, and MPO-positive cells in the penumbra zone of the ischemic cortex. **(A)** Representative imaging of double immunofluorescent labeling for YM 1/2 with CD68, iNOS with CD68 and MPO staining, counterstained with DAPI, 3 days after ischemic stroke in the ischemic penumbra. **(B–D)** Quantification of YM 1/2, iNOS, and MPO-positive cells in the ischemic penumbra. Mean ± SD, *n* = 5. Scale bar = 50 μm. *^a^P* < 0.05 vs. sham; *^b^P* < 0.05 vs. vehicle; *^c^P* < 0.05 vs. Mei.

In stroke, the initial ischemic event directly causes neuronal death. In addition, the ensuing inflammatory response induces secondary neurodegeneration, exacerbating the cell death. Neutrophil infiltration is an important process in neuroinflammation after stroke onset. Therefore, we performed immunofluorescence staining to assess neutrophil infiltration in the infarct area. Meisoindigo reduced the number of MPO^+^ (neutrophil marker) cells in the infarct area following MCAO, compared with the vehicle group (*P* < 0.05) (Figures 6A,C). MCC950 also reduced neutrophil infiltration compared with the vehicle group (*P* < 0.001) (Figures 6A,C).

**FIGURE 7:**
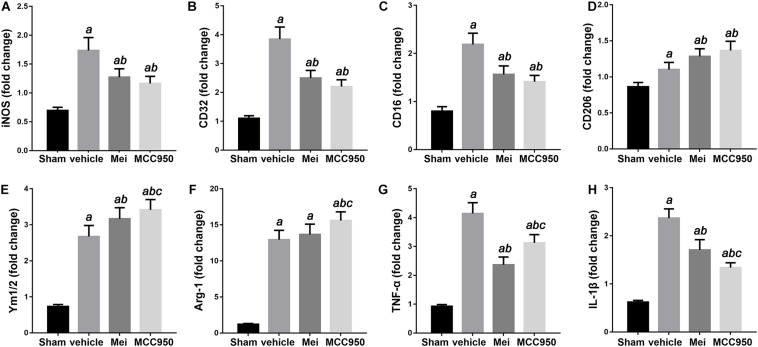
Meisoindigo injection alters mRNA expression levels of M1 and M2 polarization markers after ischemic stroke. RT-PCR was executed using total RNA extracted from ischemic cortical tissue 3 days after MCAO or from sham-operated brains. **(A–D)** Expression of mRNAs for M1 markers. **(E,F)** Expression of mRNAs for M2 markers. **(G,H)** Expression of mRNAs for TNF-α and IL-1β. Data are expressed as fold change vs. sham-operated controls. *n* = 6. *^a^P* < 0.05 vs. sham; *^b^P* < 0.05 vs. vehicle; *^c^P* < 0.05 vs. Mei.

We then examined the polarization state of microglia/macrophages using representative M1-associated (iNOS) and M2-associated (YM 1/2) markers in the ischemic hemisphere 3 days after MCAO. The number of iNOS^+^ co-localized with CD68 cells was significantly reduced in the infarct zone in mice treated with meisoindigo, and in contrast, the number of YM 1/2^+^ co-localized with CD68 cells was statistically increased, compared with the vehicle group (*P* < 0.05) (Figures 6A,B,D). In addition, we employed real-time PCR to measure the expression of polarization-related genes, including surface markers and cytokines/chemokines. The result showed that the levels of M1-type mRNAs (iNOS, CD32, and CD16) were obviuosly decreased after meisoindigo treatment (*P* < 0.05) (Figures 7A–C). However, the mRNAs of all tested M2 markers, including CD206, YM 1/2 and Arg-1, were increased after using meisoindigo injection (*P* < 0.05) (Figures 7D–F). In addition, the mRNAs expression of proinflammatory cytokines, TNF-α and IL-1β, were reduced after using meisoindigo injection (*P* < 0.05) (Figures 7G,H).

### Meisoindigo Inhibits the Activation of the TLR-4/NF-κB Signaling Pathway and Reduced Inflammatory Cytokines After Stroke

**FIGURE 8:**
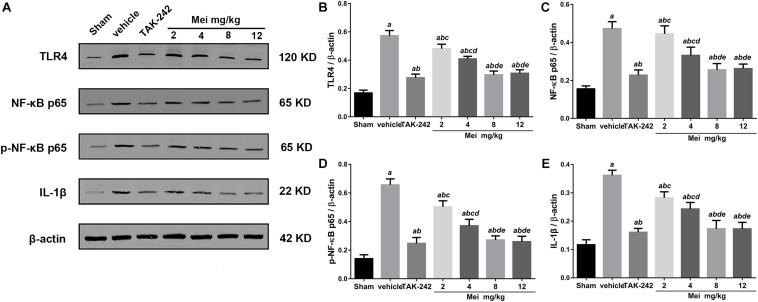
Meisoindigo suppresses the activation of the TLR-4/NF-κB signaling pathway in dose dependent manners. **(A)** Representative western blot showing that Mei treatment reduces the expression of TLR-4/NF-κB signaling pathway-related proteins in dose dependent manners. Quantitative analysis of the protein levels of TLR4 **(B)**, NF-κB p65 **(C)**, phosphorylated NF-κB p65 **(D)**, and IL-1β **(E)**. Mean ± SD; *n* = 8/group. *^a^P* < 0.05 vs. sham; *^b^P* < 0.05 vs. vehicle; *^c^P* < 0.05 vs. TAK-242; *^d^P* < 0.01 vs. 2 mg/kg Mei; *^e^P* < 0.01 vs. 4 mg/kg Mei.

**FIGURE 9:**
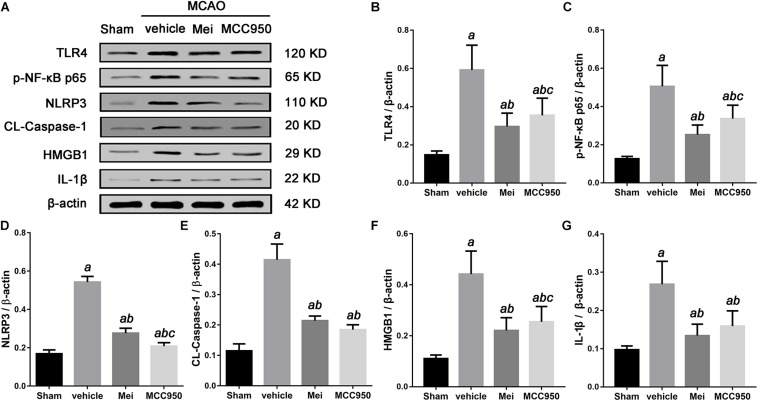
Meisoindigo treatment suppresses the activation of the TLR-4/NF-κB/NLRP3 signaling pathway after stroke. **(A)** Representative western blot showing that Mei treatment reduces the expression and phosphorylation of TLR-4/NF-κB/NLRP3 signaling pathway-related proteins. Quantitative analysis of the protein levels of TLR4 **(B)**, phosphorylated NF-κB p65 **(C)**, NLRP3 **(D)**, CL-caspase-1 **(E)**, HMGB1 **(F),** and IL-1β **(G)**. Mean ± SD; *n* = 3/group. *^a^P* < 0.05 vs. sham; *^b^P* < 0.05 vs. vehicle; *^c^P* < 0.05 vs. Mei.

To explore the molecular signaling mechanism by which meisoindigo inhibits NLRP3 inflammasome activation and blocks M1 microglia polarization, we measured the expression of TLR-4/NF-κB signaling pathway, a key intracellular signaling system that plays an important role in the initiation of the immune response in the ischemic stroke brain after meisoindigo treatment. Western blot analysis showed that TLR-4 and phosphorylated NF-κB (p-NF-κB) were elevated at 3 days after stroke in penumbra region, and their elevations were significantly inhibited by meisoindigo (*P* < 0.05) (Figure 8). Furthermore, we confirmed the directly inhibitory effect of meisoindigo on TLR-4/NF-κB pathways and found that administration of meisoindigo by different concentrations of 2, 4, 8, 14 mg/kg markedly downregulated the protein expressions of TLR4, NF-κB p65, p-NF-κB p65 and IL-1β in dose dependent manners, and had no statistical difference to compared with TAK-242, a specific inhibitor of TLR4 (*P* < 0.001) (Figure 8). Therefore, it was indicated that CIRI-induced recruitment of TLR4/NF-kB signaling pathway was probably restrained by meisoindigo (*P* < 0.05) (Figure 8) It has known that NLRP3 inflammasome acts downstream of TLR4 signaling, and TLR4/NF-κB signaling pathway plays a critical role in the activation of the NLRP3 inflammasome and in the inflammatory reaction after cerebral ischemia (Gao et al., 2009; Vartanian and Stenzel-Poore, 2010). We then performed western blot analysis of cerebral protein extracts to assess the expression of NLRP3 inflammasome-associated proteins, including NLRP3, ASC, CL-caspase-1, and inflammatory cytokines which were activated by the TLR4 pathway (*P* < 0.05) (Figure 9). The results showed that along with downregulation of TLR4 and p-NF-κB p65 by treatment of meisoindigo, the expressions of NLRP3, CL-Caspase1, HMGB1, and IL-1β were also decreased compared with the vehicle group (*P* < 0.05) (Figure 9), which had similar effect as MCC950, a specific NLRP3 inhibitor (Figure 9). Overall, our study demonstrated that meisoindigo treatment could effectively inhibit TLR4 pathway which mediated inflammasome activation, then decreased inflammatory cytokines release in a mouse MCAO model.

### Meisoindigo Increased Cell Viability After OGD/R and Inhibited NLRP3 Inflammasome-Associated Proteins Expression

**FIGURE 10:**
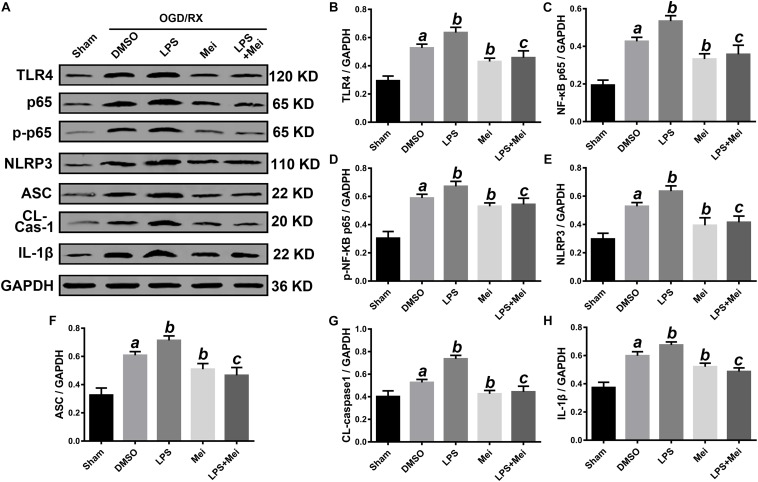
Meisoindigo treatment inhibits NLRP3 inflammasome activation through down-regulation of TLR4 pathways after OGD/R in HT-22. **(A)** Representative Western blot membranes for TLR4, NF-κB p65, phosphorylated NF-κB p65, NLRP3, ASC, CL-caspase-1, and IL-1β. Quantitative analysis of TLR4 **(B)**, NF-κB p65 **(C)**, phosphorylated NF-κB p65 **(D)**, NLRP3 **(E)**, ASC **(F)**, CL-caspase-1 **(G)**, and IL-1β **(H)** proteins expression levels. Mean ± SD; *n* = 3/group. *^a^P* < 0.05 vs. sham; *^b^P* < 0.05 vs. vehicle + OGD/R; *^c^P* < 0.05 vs. LPS + OGD/R.

**FIGURE 11:**
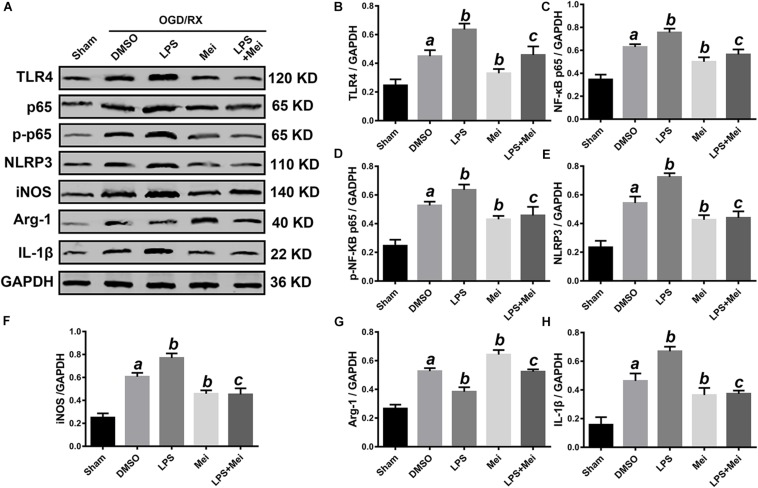
Meisoindigo treatment suppresses NLRP3 inflammasome activation and M1/M2 polarization via inhibition of TLR4 pathways after OGD/R in BV2. **(A)** Representative western blot membranes for TLR4, NF-κB p65, phosphorylated NF-κB p65, NLRP3, iNOS, Arg-1, and IL-1β. Quantitative analysis of TLR4 **(B)**, NF-κB p65 **(C)**, phosphorylated NF-κB p65 **(D)**, NLRP3 **(E)**, iNOS **(F)**, Arg-1 **(G)**, and IL-1β **(H)** proteins expression levels. Mean ± SD; *n* = 3/group. *^a^P* < 0.05 vs. sham; *^b^P* < 0.05 vs. vehicle + OGD/R; *^c^P* < 0.05 vs. LPS + OGD/R.

We then conducted *in vitro* OGD/R experiments to confirm the neuroprotective effect of meisoindigo on CIRI. Firstly, we performed CCK8 cytotoxicity assay to determine the optimal concentration of meisoindigo in the HT-22 and BV2. Then, cell viability was evaluated. The results showed that IC50 of BV2 was 30 uM and IC50 of HT-22 was 50 uM, respectively, Which we choose for subsequent experiments (*P* < 0.05) (Supplementary Figures S2C,D). Next, we performed OGD/R in HT-22 and BV2, and found that the suitable time of OGD is 6 h in BV2, and is 8 h in HT-22 (Supplementary Figures S2A,B). Then, cells were treated with meisoindigo by above selected concentration. We found that meisoindigo effectively improved the cell viability after OGD/R both in HT-22 and BV2 (*P* < 0.05) (Supplementary Figures S2E,F). Furthermore, we applied western-blot to detect the expression level of NLRP3 inflammasome-associated proteins and M1/M2 polarization, the results showed that meisoindigo significantly decreased the expression of NLRP3, ASC, CL-caspase-1 and IL-1β in the HT-22 and BV2 cells OGD/R models, which was consistent with the *in vivo* results (*P* < 0.05) (Figures 10, 11). At the same time, iNOS, a marker of M1 was downregrelated, while Arg-1, a marker of M2 was upregulated by meisoindigo treatment (*P* < 0.05) (Figure 11).

### Meisoindigo Interdicted NLRP3 Inflammasome Activation and M1/M2 Polarization Through Down-Regulation of TLR4 Pathways After OGD/R *in vitro*

Based on the above results, next, we confirmed whether TLR4 is a major molecular target of meisoindigo. We applied the LPS, a special agonist of TLR4 signaling, to further verify this hypothesis. The suitable time of OGD and greatest dose of meisoindigo in HT-22 and BV2 were determined by CCK-8 kits (Supplementary Figure S2). Compared to the OGD/R group, the expression of TLR4 and its downstream target NF-κB p65 was increased after LPS treatment both in HT-22 and BV2 (*P* < 0.01) (Figures 10, 11). However, their expressions were downregulated by co-treatment with LPS and Meisoindigo, compared with LPS only. We further detected the expressions of NLRP3 inflammasome-associated proteins, including NLRP3, ASC, CL-caspase-1 and IL-1β, which was mediated by TLR4/NF-κB pathway and upregulated after LPS stimulation, were also inhibited by co-treatment LPS and Meisoindigo both in HT-22 and BV2 (*P* < 0.05) (Figures 10, 11). The results mean that meisoindigo had great inhibitory effects on inflammatory activation in both hippocampal neurons (HT-22) and microglia (BV2) via suppressing TLR4/NF-kB pathway. In addition, we found that LPS stimulation resulted in M1 polarization of microglia (BV2) after OGD/R, but co-treatment of meisoindigo with LPS could significantly promoted the M1 microglia shifting to M2 microglia (*P* < 0.05) (Figure 11). These results strongly indicate that protective effect of meisoindigo on CIRI through inhibiting NLRP3 inflammasome and blocking M1 polarization may occur via downregulation of the TLR4 pathway.

## Discussion

Many phytochemicals have been shown to have neuroprotective effects in various neurological diseases. Meisoindigo, a traditional Chinese medicine, is derived from indirubin. However, meisoindigo does not have the toxicity of indirubin, and has been used clinically for treating CML (Ji and Zhang, 1985; Liu et al., 2001). A number of studies have shown that meisoindigo has therapeutic potential in many diseases, including inflammatory disease (Zhang et al., 2013) and cancer (Mingxin et al., 2008). In our previous study, we showed that meisoindigo inhibits leukocyte chemotactic migration in zebrafish. This led us to conjecture that meisoindigo might protect against cerebral ischemic injury. Here, we show, for the first time, that meisoindigo has neuroprotective effects after CIRI both *in vivo* and *in vitro* experiments. We measure the production of NLRP3 inflammasome and polarization of microglia/macrophages in MCAO mice treated by meisoindigo or MCC950, a selective inhibitor of the NLRP3 inflammasome, and find that MCC950 shifts microglia/macrophages from the pro-inflammatory M1 to the tissue-reparative and anti-inflammatory M2 phenotype 3 days after ischemic stroke. We then demonstrate that meisoindigo also has an anti-inflammatory effect by inhibiting NLRP3 inflammasome activation and by inducing a M1 phenotypic switch to M2 in microglia/macrophages, which has similar effect to MCC950. Importantly, we for the first time find that meisoindigo treatment can significantly inhibit the activation of TLR4/NF-κB pathway in a dose-dependent manner. Furthermore, *in vitro* study confirms that meisoindigo can significantly increase the neuronal and microglial cells viability, and inhibit neuronal and microglial NLRP3 inflammasome activation, as well as microglial M1 polarization via inhibition of TLR4/NF-κB signaling after ischemic injury, then reduced the inflammatory response secondary to reperfusion.

The inflammatory response that follows the initial ischemic insult is a key mechanism of secondary degeneration (Jin et al., 2010; Hamzei Taj et al., 2016). The critical roles of the NLRP3 inflammasome during stroke have been documented in many studies (Lepore et al., 2018; Yang J. et al., 2018; Yang X. et al., 2018; Tan et al., 2019). NLRP3 inflammasome have been demonstrated to significantly increase brain injury and neuroinflammation after stroke (Ma et al., 2018; Jing et al., 2019). In the present study, we examine the expression of NLRP3 inflammasome-associated proteins, including NLRP3, ASC, CL-caspase-1 and IL-18, to assess inflammasome activity. We find that ischemic stroke initiates NLRP3 inflammasome activation, which is inhibited by meisoindigo treatment. To further support the effect of meisoindigo on inhibition of NLRP3 activation and clarify the relationship between NLRP3 inflammasome and microglia/macrophages polarization, specific inhibitors of NLRP3, MCC950 is utilized. MCC950 also markedly downregulates NLRP3 inflammasome markers to an extent similar to meisoindigo. We also find that microglia/macrophages are clearly shifted from the pro-inflammatory M1 phenotype toward the anti-inflammatory M2 phenotype, recognized as the upregulation of the M2-associated marker and the downregulation of the M1-associated marker by immunofluorescence and QPCR, by MCC950 treatment 3 days after MCAO. These findings suggest that the NLRP3 inflammasome may induce the polarization of classically-activated M1 microglia/macrophages, and increase the secretion of pro-inflammatory cytokines. The NLRP3 inflammasome components activation have great promotion effects on microglia/macrophage polarization, thereby exacerbating the inflammatory response in the acute phase of ischemic stroke. Comparing the MCC950, the inhibitory effect on NLRP3 inflammasome and the function on microglia/macrophage polarization are no statistic differences in meisoindigo treatment. Thus, the neuroprotective effect of meisoindigo may be related to inhibit NLRP3 inflammasome activation and M1 microglia polarization. The inhibitory effects of meisoindigo on NLRP3 inflammasome partly contributes to its suppressing effects on M1 microglia polarization. Other mechanisms such as direct inhibition or suppression of NLRP3 inflammasome-independent ways may also be responsible for interdicting of M1 microglia polarization after meisoindigo treatment. Further and more studies are needed to clearly illuminate the mechanistic link between NLRP3 inflammasome activation and microglia/macrophage polarization.

The NLRP3 inflammasome is present in many cells. In the CNS, a great number of studies have shown that the NLRP3 inflammasome is mainly localized in microglia, although some studies suggest that NLRP3 is also present in astrocytes and neurons (Qin et al., 2018). Here, we found that NLRP3 labeling colocalized more with NeuN (neuronal marker) labeling than with CD68 (microglia/macrophage marker) labeling after 3 days of reperfusion. Hu et al. (2012) suggested that neurons shed their components and/or released soluble factors to drive the M2–M1 shift. We then speculate that following stroke, the NLRP3 inflammasome in neurons is activated and that pro-inflammatory cytokines are released, which in turn may modulate microglia/macrophage polarization. The activation of microglia/macrophages may relate to NLRP3 inflammasome activation in neurons. Meisoindigo treatment significantly suppresses NLRP3 inflammasome activation, increases the number of surviving neurons, and reduces the activation and infiltration of CD68^+^ microglia/macrophages in the penumbra 3 days after MCAO. However, further study is needed to test this conjecture.

Recent studies show that microglia/macrophages play dual, contrasting, roles in neuronal injury and recovery, which may be related to their ability to polarize into the M1 or M2 phenotype (Liu et al., 2013; Hamzei Taj et al., 2016). Microglia/macrophages dynamically respond to microenvironmental changes during the progression of ischemic injury. In the acute phase, M2-dominant microglia/macrophages make up the vast majority of recruited leukocytes and provide protective functions. However, 1 week following injury, the M1 phenotype predominates (Kigerl et al., 2009; Wang et al., 2013). A defect in M2-inducing endogenous signals has been shown to exacerbate cerebral ischemia (Girard et al., 2013). Therefore, strategies that promote the shift from the M1 to the M2 phenotypic state may have therapeutic potential for cerebral ischemia. Notably, we discovery that meisoindigo promotes the shift from the destructive M1 state to the neuroprotective and tissue-repairing M2 state in the ischemic brain. One possible mechanism may be that meisoindigo inhibits NLRP3 inflammasome activation both in neurons and microglia/macrophages. When the NLRP3 inflammasome of neurons is inactivated by meisoindigo, neuronal pyroptosis is reduced (Alishahi et al., 2019), and the pro-inflammatory cytokines such as IL-18, IL-1β and IL-6 are unreleased, then the exogenous stimulant derived from neurons on infiltration, activation and polarization of microglia/macrophage is decreased. In addition, meisoindigo at the same time inhibits NLRP3 inflammasome activation of microglia/macrophage themselves, also blocks their endogenous initiate of infiltration, activation and polarization. There are two ways including canonical and non-canonical pathways participated in the activation of NLRP3 inflammasome. In the canonical pathways, TLR4/NF-κB pathways activation is fundamental step of the NLRP3 inflammasome formation (Zhong et al., 2019). Thus, to explore possible pathways involving in meisoindigo-mediated neuroprotective action in ischemic stroke, we focus on TLR signaling pathway. Our study demonstrated the meisoindigo had neuroprotective effects on cerebral ischemia through decreasing NLRP3 inflammasome components expression via inhibition of activation of the TLR4/NF-κB pathways. The TLR, a pattern-recognition receptor that detects microbial components, plays a major role in the initiation of the immune response (Rau et al., 2018). The TLR signaling pathway is involved in removing bacteria, but also have damaged effects on brain cells. Activation of TLR4, which is expressed in microglia, astrocytes and neurons in the brain, is one of the imitates of NLRP3 inflammasome activation and is involved in the release of inflammatory mediators (such as IL-1, IL-6, TNF-α, COX-2 and iNOS) by activating the NF-κB signaling pathway in macroglia, astrocytes, and neurons (Okun et al., 2011; Parajuli et al., 2012; Garate et al., 2014; Badshah et al., 2016). TLR4 and the downstream target, NF-κB are found to facilitate the inflammatory reaction in cerebral ischemia and infarction (Zheng et al., 2017) We test whether TLR4/NF-κB is involved in the inhibitory effect of meisoindigo on NLRP3 inflammasome activation and microglia/macrophage polarization, and firstly find that meisoindigo obviously decreases the stroke-induced upregulation of TLR4/NF-kB pathway proteins and IL-1β in concentration-dependent manners, which has no statistic difference by comparing with treatment of TAK-242, a specific inhibitor of TLR4. Furthermore, in *in vitro* OGD/R experiments, we find that meisoindigo also blocks the activation of TLR4/NF-kB signaling pathway induced by LPS stimulation in neurons and microglia. Moreover, we show that, at the same time, along with the inactivaion of TLR4/NF-kB, the expression of NLRP3 inflammasome-associated proteins is decreased and the shifting from M1 microglia to M2 microglia is increased *in vivo* and *in vitro*. All these finds suggest that meisoindigo may exert neuroprotection in the ischemic brain by partly suppressing the TLR4/NF-κB/NLRP3 inflammasome, subsequently blocking M1 microglia polarization. In addition, we think that meisoindigo switching microglia to M2 phenotype may also attribute to inhibition of TLR4/NF-κB signaling pathway only.

**FIGURE 12:**
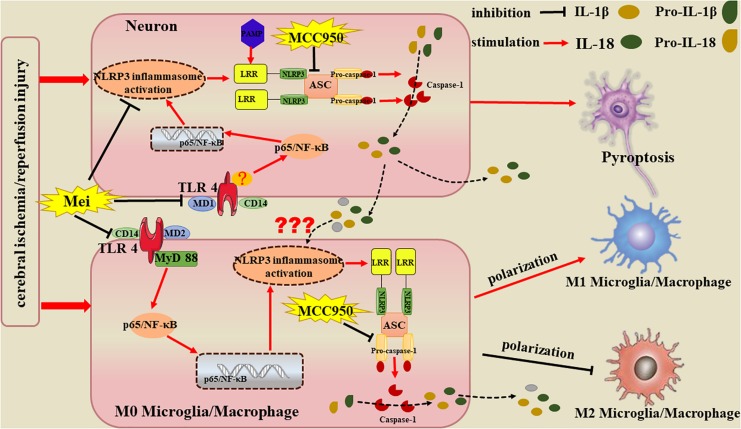
Potential mechanisms by which Meisoindigo protects against focal cerebral ischemia-reperfusion injury (CIRI). Activation of the NLRP3 inflammasome and microglia/macrophage polarization strongly influence the immunological cascades triggered by stroke. Meisoindigo reduces the production of inflammatory mediators by inhibiting the activation of the NLRP3 inflammasome and preventing the microglial/macrophage switch from the protective M2 phenotype to the pro-inflammatory M1 phenotype, thereby exerting neuroprotection against ischemic stroke, which may achieve through the inhibition of TLR4/NF-κB signaling pathway in neurons and microglia. In addition, we here found that the NLRP3 inflammasome is mainly expressed in neurons. Therefore, we speculate that the inflammatory mediators released by NLRP3 inflammasome1 activation in neurons may induce neuronal pyroptosis and affect phenotypic polarization in microglia/macrophages.

Overall, Meisoindigo has neuro-protective effect on ischemic brain, the protection attributes to its ability of relieving the brain inflammation by inhibiting the activation of the NLRP3 inflammasome and preventing the microglial/macrophage switch from the pro-inflammatory M1 phenotype to the protective M2 phenotype, which may obtain through the inhibition of TLR4/NF-κB signaling pathway in neurons and microglia. In addition, we presently show that the NLRP3 inflammasome is mainly existed in neurons after stroke, suggesting pro-inflammatory cytokines such as IL-18, IL-1β and IL-6 are released due to neuronal NLRP3 inflammasome activation, which, presumably, exogenously stimulates the activation and M1 polarization of microglia/macrophages, except for endogenous activation by their own NLRP3 inflammasome (Figure 12). Meisoindigo and other traditional Chinese medicines may function as physiological modulators. By inhibiting the activation of the NLRP3 inflammasome and by regulating M1/M2 polarization, meisoindigo is able to exert a wide variety of protective effects. The efficacy of meisoindigo in models of ischemic brain injury might be attributed to its pleiotropic actions. Further study is needed to clarify the comprehensive mechanisms of the phenotype-modulating and neuroprotective properties of meisoindigo at cellular and molecular level.

## Data Availability Statement

The datasets generated and/or analyzed during the current study are available from the corresponding author on reasonable request in compliance with ethical standards.

## Ethics Statement

The study was approved by the Ethics Committee of Renmin Hospital of Wuhan University (No. WDRM-20170504) and performed in compliance with the ARRIVE guidelines.

## Author Contributions

YY carried out all the *in vitro* works and made all the figures. TJ performed all the animal surgeries, the tissue preparation, and a part of the Western Blot. XZ and BY conducted the experiments on mRNA expression levels of M1 and M2 polarization markers. ZZ conducted the *in vitro* experiments. JW assisted with the immunostaining. YZ supported the tissue preparation and brain tissue isolation. XX participated in the experimental design, the manuscript preparation, and the final approval of the manuscript. LG conceived and designed the project, supervised the work, and prepared and approved the manuscript. All authors read and approved the final manuscript.

## Conflict of Interest

The authors declare that the research was conducted in the absence of any commercial or financial relationships that could be construed as a potential conflict of interest.

## Footnotes

1 https://pga.mgh.harvard.edu/primerbank/

## Supplementary Material

FIGURE S1 The survival rate and death rate of different groups within 3 days post MCAO. **(A)** The survival rate of mice in different groups. **(B)** The death rate in different groups. Data represent the mean ± SD (*n* = 12). *^a^P* < 0.05 vs. Sham; *^b^P* < 0.05 vs. vehicle, *^c^P* < 0.05 vs. Mei 4 mg/kg.

FIGURE S2 Meisoindigo increased cell viability after OGD in both BV2 and HT-22. **(A,B)** After suffering different time of deprived oxygen-glucose, the suitable time of OGD is 6 h in BV2, and is 8 h in HT-22. **(C,D)** The IC50 of BV2 is 30 uM and the IC50 of HT-22 is 50 uM. **(E,F)** Cell viability following different concentrations of meisoindigo after OGD. Data represent the mean ± SD (*n* = 3). *^∗^P* < 0.05 vs. 0 h OGD group, *^#^P* < 0.05 vs. 0 uM Mei group, *^a^P* < 0.05 vs. OGD group.
