# Supplementary figures and images for: Meisoindigo Protects Against Focal Cerebral Ischemia-Reperfusion Injury by Inhibiting NLRP3 Inflammasome Activation and Regulating Microglia/Macrophage Polarization via TLR4/NF-κB Signaling Pathway
Source: Front Cell Neurosci. 2019 Dec 16;13:553. doi: 10.3389/fncel.2019.00553 (PMC6930809; doi:10.3389/fncel.2019.00553)

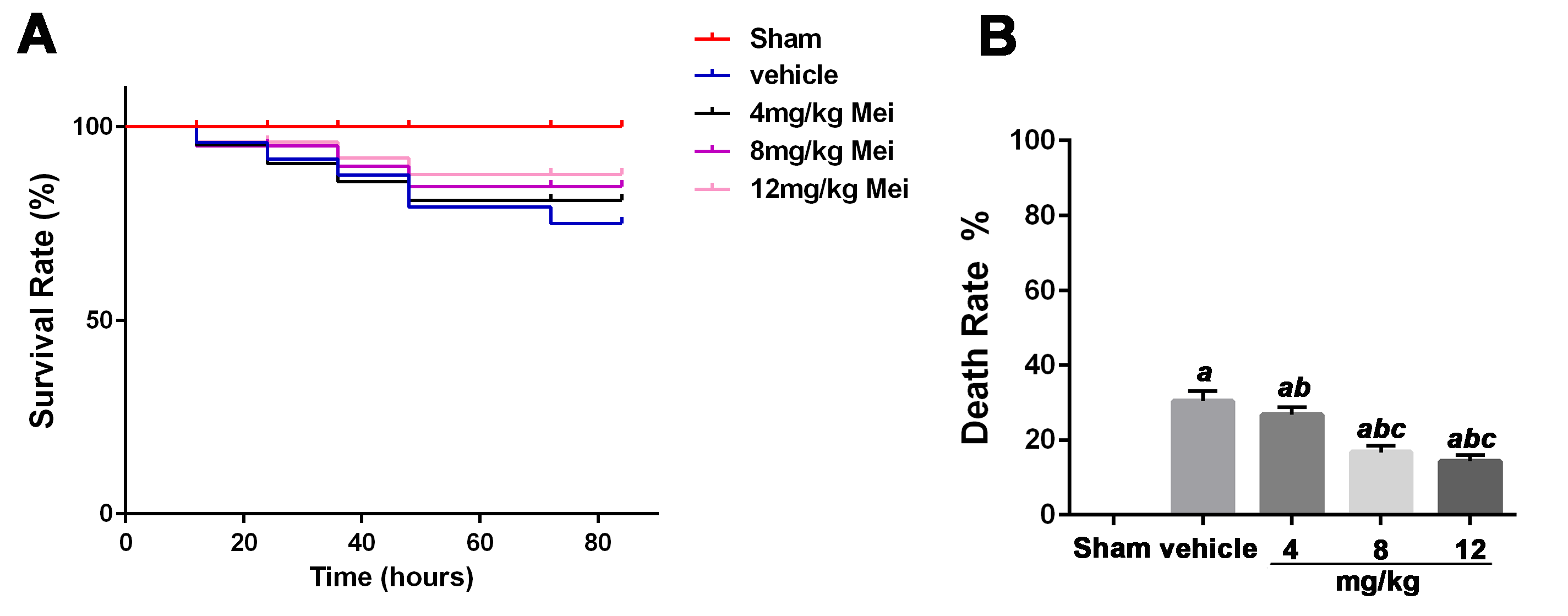

Supplement: FIGURE S1 — The survival rate and death rate of different groups within 3 days post MCAO. (A) The survival rate of mice in different groups. (B) The death rate in different groups. Data represent the mean ± SD (n = 12). aP < 0.05 vs. Sham; bP < 0.05 vs. vehicle, cP < 0.05 vs. Mei 4 mg/kg. [file Image_1.TIF]

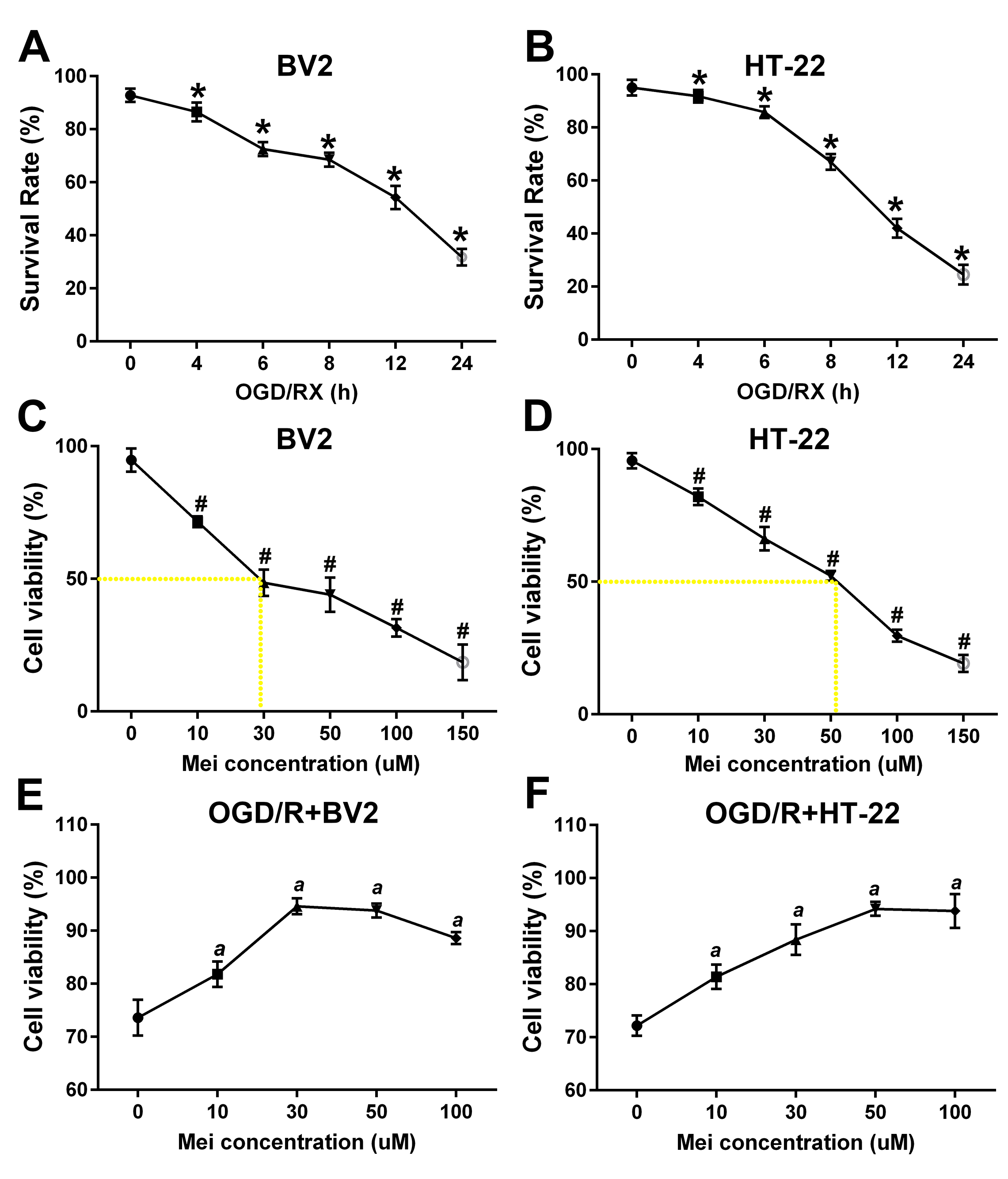

Supplement: FIGURE S2 — Meisoindigo increased cell viability after OGD in both BV2 and HT-22. (A,B) After suffering different time of deprived oxygen-glucose, the suitable time of OGD is 6 h in BV2, and is 8 h in HT-22. (C,D) The IC50 of BV2 is 30 uM and the IC50 of HT-22 is 50 uM. (E,F) Cell viability following different concentrations of meisoindigo after OGD. Data represent the mean ± SD (n = 3). ∗P < 0.05 vs. 0 h OGD group, #P < 0.05 vs. 0 uM Mei group, aP < 0.05 vs. OGD group. [file Image_2.TIF]
